# Eight week exposure to a high sugar high fat diet results in adiposity gain and alterations in metabolic biomarkers in baboons (*Papio hamadryas *sp.)

**DOI:** 10.1186/1475-2840-9-71

**Published:** 2010-10-29

**Authors:** Paul B Higgins, Raul A Bastarrachea, Juan Carlos Lopez-Alvarenga, Maggie Garcia-Forey, J Michael Proffitt, V Saroja Voruganti, M Elizabeth Tejero, Vicki Mattern, Karin Haack, Robert E Shade, Shelley A Cole, Anthony G Comuzzie

**Affiliations:** 1 Department of Genetics, Southwest Foundation for Biomedical Research, San Antonio, Texas, USA; 2 Southwest National Primate Research Center, San Antonio, Texas, USA; 3 Hospital General de Mexico, Mexico City, Mexico; 4 INMEGEN, Mexico City, Mexico; 5 Southwest Foundation for Biomedical Research, San Antonio, Texas, USA

## Abstract

**Background:**

Baboons (*Papio hamadryas *Sp.) develop features of the cardiometabolic syndrome and represent a clinically-relevant animal model in which to study the aetiology of the disorder. To further evaluate the baboon as a model for the study of the cardiometabolic syndrome, we developed a high sugar high fat diet and hypothesized that it could be used to induce adiposity gain and affect associated circulating biomarkers.

**Methods:**

We developed a diet enriched with monosaccharides and saturated fatty acids that was composed of solid and liquid energy sources. We provided a group of baboons (n = 9) *ad libitum *access to this diet for 8 weeks. Concurrently, a control group (n = 6) was maintained with *ad libitum *access to a low sugar low fat baseline diet and normal water for 8 weeks. Body composition was determined by dual-energy X-ray absorptiometry and circulating metabolic biomarkers were measured using standard methodology before and after the 8 week study period.

**Results:**

Neither body composition nor circulating biomarkers changed in the control group. Following the 8 weeks, the intervention group had a significant increase in fat mass (1.71 ± 0.98 *vs*. 3.23 ± 1.70 kg, p = 0.004), triglyceride (55 ± 13 *vs*. 109 ± 67 mg/dL, p = 0.006,), and leptin (1.19 ± 1.40 *vs*. 3.29 ± 2.32 ng/mL, p = 0.001) and a decline in adiponectin concentrations (33530 ± 9744 *vs*. 23330 ± 7863 ng/mL, p = 0.002). Percentage haemoglobin A_1C _(4.0 ± 0.3 *vs*. 6.0 ± 1.4, p = 0.002) also increased in the intervention group.

**Conclusions:**

Our findings indicate that when exposed to a high sugar high fat diet, young adult male baboons develop increased body fat and triglyceride concentrations, altered adipokine concentrations, and evidence of altered glucose metabolism. Our findings are in keeping with observations in humans and further demonstrate the potential utility of this highly clinically-relevant animal model for studying diet-induced metabolic dysregulation.

## Background

The cardiometabolic syndrome represents one of the most significant current global public health issues [1]. Manifestations of the cardiometabolic syndrome include increased central adiposity, insulin resistance, and dyslipidaemia [2]. Collectively these and other related abnormalities represent an increased risk for the development of chronic diseases such as type 2 diabetes and coronary heart disease [3]. The aetiology of the cardiometabolic syndrome is not completely understood. Studies in humans and in animal models have suggested that exposure to Western diets- particularly those containing sugar-sweetened beverages, contribute to its development [4-8]. The mechanisms underlying the contribution of diet to the cardiometabolic syndrome have yet to be fully elucidated.

Efforts to determine the mechanisms underlying the dietary inducement of the cardiometabolic syndrome could be enhanced through the use of more clinically-relevant animal models. The baboon (*Papio hamadryas *sp.) is an established non-human primate model with a long history in biomedical research [9,10]. Baboons are a long-lived old world monkey species (*Catarhini*) known to consume diets containing plants, fruits, and some animal matter [11,12]. Wild baboons feeding on human waste foods manifest metabolic abnormalities consistent with human obesity and the cardiometabolic syndrome [13,14]. Our previous work has shown that captive baboons are susceptible to obesity even when maintained on a diet low in fat and simple carbohydrates [15]. We, along with others, have also described impaired insulin signalling at the whole body and molecular level [16] and, in some cases overt diabetes [17] in spontaneously obese baboons.

To further evaluate the potential of the baboon model for the study of diet-induced cardiometabolic syndrome, we developed an experimental diet with a fatty acid and monosaccharide composition similar to that of a typical fast food diet. We hypothesized that we could induce adiposity gain and influence associated metabolic biomarkers using this diet. Herein, we report changes in fat mass, adipokines, triglyceride, and percentage haemoglobin A_1C _(%HbA_1C_) in baboons after only 8 weeks of *ad libitum *consumption of this diet.

## Methods

### Animals

Fifteen male baboons (*Papio hamadryas Sp*.) from the Southwest National Primate Research Center (San Antonio, TX) colony were studied. All animals were group-housed in outdoor enclosures according to established National Research Council guidelines. To minimize disruption to social rankings and normal behaviours, two established groups of animals were selected for the study. A Veterinarian performed standard health assessments, including blood chemistry and hematologic profiles, on all animals before the start of the study. Animals had no prior significant medical history. All study procedures were approved by the Institutional Animal Care and Use Committee of the Southwest Foundation for Biomedical Research, San Antonio, TX.

### Design

At baseline all animals had been consuming the commercially available 5LE0 solid feed (LabDiet, PMI, St. Louis, MO) *ad libitum*. This feed is high in complex carbohydrates and low in fat. Animals also had *ad libitum *access to normal water (Table 1). Following a 12 hour overnight fast, animals were sedated with ketamine HCl (10 mg/Kg) before undergoing baseline body composition assessment and collection of blood samples. Each group was then randomly assigned to receive one of two diets for 8 weeks:

**Table 1:** Energy composition of diets

	Energy density	%Kcal as fat	%Kcal as carbohydrate	%Kcal as sugar	%Kcal as protein
5LE0 solid chow^1^	3.26^3^	13.81	67.16	2.95	19.03
High fat chow^2^	4.33^3^	38.46	51.00	10.72	10.53
Sweetened water^1^	0.42^4^	0	100.00	100.00	0

^1^From chemical analysis of macronutrient composition provided by manufacturer, ^2^from chemical analysis of nutrient composition from Covance Laboratories (Madison, WI), ^3^Kcal/g, ^4^Kcal/mL.

1) Control group (n = 6) - continued *ad libitum *access to the low sugar low fat baseline diet and normal water; and

2) Intervention group (n = 9) - *ad libitum *access to the palatable high sugar high fat experimental diet containing solid and liquid energy sources and normal water.

Twelve-hour fasted blood samples and body composition assessments were again collected after 8 weeks of dietary exposure.

### Experimental diet

Preliminary experiments were undertaken on singly-housed baboons to determine the palatability of various high saturated fatty acid solid feed preparations. The feed found to be most palatable was prepared using 73% Purina Monkey Chow 5038 (a grain-based meal), 7% lard, 4% Crisco, 4% coconut oil, 10.5% flavoured high fructose corn syrup, and1.5% water. Vitamins and mineral preparations were added to match the micronutrient composition of the baseline feed. Palatability was enhanced using non-caloric artificial fruit flavours and by lightly baking the feed for 12 minutes at 300°F. Liquid calories were offered to the intervention group in a beverage that was prepared using water, high fructose corn syrup (2.83 Kcal/g, 76% sugar, 41.8% fructose, 34.2% dextrose, ISOSWEET 5500, Staley, Decatuer, IL), and artificial fruit flavouring. The sweetened water contained 110 mL of high fructose corn syrup per litre. A summary of the nutritional composition of the solid feed and the sweetened water is given in Table 1.

### Body composition

Body weight was measured on an electronic scale (GSE 665, Texas Scales Inc., Cibolo, TX). Dual energy X-ray absorptiometry (DXA) body composition scans were undertaken using a Lunar Prodigy densitometer (GE Healthcare, Madison, WI). Animals were placed in the supine position on the DXA bed and extremities were positioned within the scanning region. Scans were analyzed using encore2007 software version 11.40.004 (GE Healthcare, Madison, WI). Total body, trunk region (torso), and limb (arm + leg) region compositions were determined. The coefficients of variation for total fat mass and total lean mass for two replicate scans in 3 baboons were found to be 2.2 and 2.3%, respectively.

### Glucose, %HbA_1c_, serum lipid, and lipoprotein cholesterol measurements

Fasting plasma concentrations of glucose, triglyceride, total cholesterol, LDL cholesterol, and HDL-cholesterol were determined using an ACE clinical analyzer (Alfa Wasserman Diagnostic Technologies LLC; West Caldwell, NJ). Total haemoglobin and %HbA_1c _were determined from whole blood using the ACE clinical analyzer.

### Plasma insulin, C-reactive protein, and adipokine measurements

Plasma insulin, leptin, and total adiponectin concentrations were determined using commercially available radioimmunoassay kits [Millipore Inc., Billerica, MA]; the intra-assay CVs were 2.2%., 2.2%, and 5.2%, respectively. Plasma C-reactive protein was measured using the Monkey C-reactive protein ELISA kit [Life Diagnostics Inc., West Chester, PA].

### Statistical analyses

Data from each diet group was analyzed independently; pre- and post-intervention means were evaluated using two-tailed paired samples *t*-tests. Variable values were converted to common logarithms to correct for deviations from the normal distribution when necessary. Statistical significance was set at *P *< 0.05. Statistical analyses were undertaken using SYSTAT version12 [Chicago, IL].

## Results

Basic descriptive characteristics of the study animals are presented in Table 2. All animals passed physical examinations and had normal blood chemistry and hematologic profiles at the study outset. None of the groups had a statistically significant change in body weight, although weight change did approach statistical significance in the intervention group (p = 0.09).

**Table 2:** Descriptive statistics

	Controls (n = 6)	Intervention (n = 9)
Age at baseline (yrs.)	7.4 ± 0.3	7.8 ± 0.5
Baseline weight (Kg)	26.6 ± 2.8	29.0 ± 3.0
8-week weight (Kg)	26.5 ± 2.4	30.1 ± 3.3

Data are mean ± SD. Controls had continued *ad libitum *access to the baseline diet and water. Intervention group had *ad libitum *access to the experimental diet.

No differences were observed between baseline and 8 week body composition and metabolic variables in the control group (Table 3). Animals in the intervention group gained fat mass, trunk fat mass, and percentage fat mass (Table 4). The proportion of total fat mass gain that occurred in the trunk region was 75.5 ± 31.5%. Triglyceride, leptin, and %HbA_1C _also increased significantly from baseline in this group (Table 4). In addition, adiponectin concentrations decreased significantly after the 8 week feeding period. Fasting glucose, insulin, C-reactive protein, total-, LDL-, and HDL-cholesterol concentrations were not significantly affected (Table 4).

**Table 3:** Body composition and metabolic variables at baseline and after 8-weeks in the control group

	Baseline	8 weeks	p value
Total body fat mass (Kg)	1.50 ± 0.68	1.46 ± 0.37	0.74
Total body lean mass (Kg)	23.5 ± 2.3	23.2 ± 2.3	0.68
Percentage fat mass	5.7 ± 2.1	5.6 ± 1.2	0.70
Trunk fat mass (Kg)	0.89 ± 0.56	0.89 ± 0.28	0.93
Trunk lean mass (Kg)	12.3 ± 1.4	12.2 ± 1.4	0.91
Limb fat mass (g)	453 ± 102	426 ± 77	0.13
Limb lean mass (kg)	8.9 ± 1.1	8.9 ± 1.0	0.36
Bone mineral content (g)	1104 ± 69	1095 ± 107	0.66
Fasting glucose (mg/dL)	88.8 ± 3.4	97.0 ± 10.7	0.20
Fasting insulin (μU/mL)	11.4 ± 4.3	25.4 ± 23.4	0.34
%Hemoglobin A_1c_	4.4 ± 0.3	5.7 ± 2.7	0.21
Total cholesterol (mg/dL)	91.2 ± 15.6	85.7 ± 15.7	0.27
LDL cholesterol (mg/dL)	35.7 ± 10.7	34.2 ± 9.3	0.67
HDL cholesterol (mg/dL)	44.2 ± 10.6	40.3 ± 9.1	0.29
Triglyceride (mg/dL)	55.5 ± 10.3	55.8 ± 15.0	0.89
Leptin (ng/mL)	0.99 ± 0.42	1.06 ± 0.23	0.58
Adiponectin (ng/mL)	29556 ± 4153	27930 ± 5505	0.13
C-reactive protein (ng/mL)	44.8 ± 30.6	47.2 ± 42.0	0.80

Data are mean ± SD. Controls had continued *ad libitum *access to the baseline diet and water. Means comparisons were made using paired *t*-tests. Variables were log_10 _transformed before statistical analyses were undertaken, n = 6.

**Table 4:** Body composition and metabolic variables at baseline and after 8-weeks in the intervention group

	Baseline	8 weeks	p value
Total body fat mass (Kg)	1.71 ± 0.98	3.23 ± 1.70	0.004
Total body lean mass (Kg)	25.7 ± 2.7	24.8 ± 2.6	0.003
Percentage fat mass	5.9 ± 3.3	10.8 ± 5.4	0.002
Trunk fat mass (Kg)	1.04 ± 0.84	2.38 ± 1.45	0.004
Trunk lean mass (Kg)	13.3 ± 1.3	12.9 ± 1.3	0.11
Limb fat mass (g)	489 ± 132	626 ± 204	0.02
Limb lean mass (kg)	9.8 ± 1.2	9.6 ± 1.2	0.22
Bone mineral content (g)	1237 ± 122	1242 ± 124	0.72
Fasting glucose (mg/dL)	95.4 ± 16.6	87.6 ± 5.1	0.22
Fasting insulin (μU/mL)	18.4 ± 16.1	18.9 ± 8.8	0.95
%Hemoglobin A_1c_	4.0 ± 0.3	6.0 ± 1.4	0.002
Total cholesterol (mg/dL)	103.3 ± 28.7	108.2 ± 20.9	0.22
LDL cholesterol (mg/dL)	38.0 ± 12.5	37.9 ± 14.5	0.71
HDL cholesterol (mg/dL)	55.0 ± 17.8	48.7 ± 13.7	0.42
Triglyceride (mg/dL)	55 ± 13	109 ± 67	0.006
Leptin (ng/mL)	1.19 ± 1.40	3.29 ± 2.32	0.001
Adiponectin (ng/mL)	33530 ± 9744	23330 ± 7863	0.002
C-reactive protein (ng/mL)	53.0 ± 48.8	81.5 ± 83.1	0.44

Data are mean ± SD. Intervention group had *ad libitum *access to the experimental diet and water. Means comparisons were made using paired *t*-tests. Variables were log_10 _transformed before statistical analyses were undertaken, n = 9.

## Discussion

Baboons spontaneously develop obesity, insulin resistance, dyslipidaemia, and type 2 diabetes. We hypothesized that we could induce adiposity gain and alter circulating metabolic biomarkers in baboons by exposing a group to an experimental diet designed to be representative of a typical fast food diet containing solid and liquid energy sources. The animals gained fat mass, particularly in the trunk region and had significant increases in triglyceride, leptin, and %HbA_1C_, and a decline in adiponectin concentrations. Our findings demonstrate that, in as little as 8 weeks, baboons exposed to a high sugar high fat diet gained fat mass and manifested biochemical alterations consistent with the early stages of metabolic dysregulation.

Baboons are found primarily in the East African savannah although populations are known to extend over much of the continent. They are diurnal and terrestrial animals that have been noted as indiscriminate consumers of vegetable and animal matter. Among wild baboons feeding on human foods, some were shown to have significantly greater body mass, leptin, insulin, and glucose concentrations relative to their normal diet-consuming counterparts [13]. Given the relatively recent common ancestry, baboons and humans are genetically, anatomically, and physiologically very similar. Baboons and humans also appear to develop similar metabolic disorders; earlier studies have documented spontaneous obesity, insulin resistance, and a form of adult-onset diabetes in captive baboons [15-19]. We, along with others, have described genetic effects on obesity and cardiovascular disease risk factors in keeping with observations in human populations [15,18,20]. In addition, baboons are known to manifest dyslipidemia and develop fatty arterial lesions when exposed to diets high in saturated fatty acids and cholesterol [21-23]. Combined, these observations suggest that the baboon model could prove very useful for furthering our understanding of the aetiology of the cardiometabolic syndrome.

We provided a group of young male adult baboons ad *libitum *access to a palatable high sugar high fat experimental diet for 8 weeks, while a control group of similar age and weight were maintained on the baseline low sugar low fat diet. The experimental diet was composed of a solid feed with a macronutrient composition similar to that of a typical fast food burger and French fries and a sweetened drink with a monosaccharide composition similar to that of a typical soft drink.

Body composition did not change in the control group. The intervention group gained fat mass but lost total body lean mass, and as a result, significant changes in body weight did not occur. The majority of the gain in fat mass occurred in the trunk region. This pattern is consistent with the adiposity pattern that is associated with central obesity in human males [24] and with fat gain patterns observed in moderate fat-fed dogs [25]. It is not clear why the animals lost lean body mass and we are unaware of similar findings in other overfeeding studies [26-28]. The animals had *ad libitum *access to water throughout the study period and it is unlikely that the diet had an effect on hydration status. The protein content of the experimental solid feed was less than that of the baseline feed, but would have been adequate to maintain lean mass. However, it is possible that the animals' protein intake was diminished as a significant quantity of their energy intake came from the sweetened water.

Fasting plasma glucose and insulin concentrations were not significantly affected in the intervention group. Nevertheless, mean %HbA_1c _increased by almost 70%. This apparent discrepancy may have several causes. It is possible that hepatic insulin resistance, reflected primarily by fasting glucose and insulin concentrations, had not developed after only 8 weeks. Alternatively, ketamine, used to sedate the animals before the blood draws, is known to have effects on glucose and insulin concentrations [29] and may have obscured the diet's effects on fasting glucose and insulin concentrations. The acute changes in glucose resulting from the ketamine sedation would not have affected %HbA_1c_. If the former explanation is true, then the increase in glycated haemoglobin must have resulted from elevated postprandial glucose concentrations in the presence of emerging peripheral tissue insulin resistance. Direct assessments of hepatic and peripheral tissue insulin resistance would be required to address these issues. Regardless, the rise in %HbA_1c _suggests that alterations in glucose homeostasis may have developed after only 8 weeks of consuming the diet. In support, the decline in adiponectin concentrations is consistent with a worsening of glucose homeostasis [30]. Notably, these baboons had a very low body fat percentage at baseline. It is conceivable that a larger effect on glucose metabolism could have been observed if the animals had greater body fat at baseline.

We did not observe changes in cholesterol concentrations in response to the diet. Nonetheless, fasting triglyceride and leptin concentrations were markedly increased while adiponectin concentrations decreased. Earlier studies in baboons have not described triglyceride changes in animals exposed to high saturated fatty acid diets [31]. However, exposure to high monosaccharide diets have been shown to result in elevated triglycerides in this species [23]. In humans, 4 week consumption of fructose at 17% of total energy intake has been shown to increase fasting triglyceride without influencing cholesterol [32]. Several other studies have also shown triglyceride to be an early responder to high monosaccharide (particularly fructose) overfeeding in humans [33-35]. Adipokines are known to respond to dietary manipulation [36]. Elevated leptin has also been shown to be an early response to high fat and high carbohydrate overfeeding in humans and in rodents [37-39]. Similar findings have been reported for adiponectin in human studies [40]. Interestingly, the triglyceride and leptin responses of the baboons are consistent with those reported in a 4 week overfeeding study in humans in which a solution of fructose was used to deliver the excess dietary energy: fasting insulin and total cholesterol concentrations also remained unaltered in that study [41]. Hence, intake of the monosaccharide enriched sweetened water was likely to be responsible for the triglyceride response observed in this group of baboons. Because of the constraints of the outdoor housing environment, it was not possible to accurately quantify energy intake. Hence, we cannot dismiss the possibility that triglyceride and adipokine responses were attributable to increased total energy intake. This would have to be determined using another experimental design and was not the objective of this study. Nevertheless, our estimates of solid feed and drink consumption suggest that the energy intake of the treatment group was much higher than that of the control group and that a large proportion of the excess energy intake came from the sweetened water (data not shown). Regardless, the alterations in triglyceride, leptin, and adiponectin observed in baboons exposed to our diet are consistent with those seen in human overfeeding studies, particularly those that tested the effects of monosaccharide overfeeding. Taken together, the data suggest that triglyceride, leptin, and adiponectin may be early responders to excessive energy intake and may represent markers of emerging metabolic dysregulation.

Importantly, the short 8 week dietary exposure leaves additional questions, particularly those relating to whether this diet could result in, and if so the time course taken to develop, overt cardiometabolic syndrome or related end-stage diseases. The rate of fat mass accrual suggests that the time taken to become overtly obese would be rather short. Indeed, studies undertaken in the closely-related rhesus macaque species have documented an earlier onset of type 2 diabetes than in humans [42]. Future studies will be designed to address these questions.

## Conclusions

Our findings indicate that young male baboons exposed to a high sugar high fat diet, composed of solid and liquid energy sources, developed multiple metabolic abnormalities consistent with emergent cardiometabolic syndrome. Our findings further demonstrate the potential utility of this animal model for studying diet-induced metabolic dysregulation.

## List of abbreviations

DXA: dual energy X-ray absorptiometry; %HbA_1C_: percentage haemoglobin A_1C_.

## Competing interests

The authors declare that they have no competing interests.

## Authors' contributions

AGC, JCLA, RAB, and PBH conceived and designed the study. PBH wrote the manuscript. PBH, JCLA, and AGC analyzed the data. JCLA, MGC, RAB, VSV, RES, PBH, and AGC developed and conducted preliminary tests on the diet. JCLA, PBH, MGF, JMP, VSV, MET, VM, SAC, and KH participated in data collection and biochemical analyses. Manuscript edits were performed by JCLA, VSV, MET, KH, SAC, RES, and AGC. Additional consultation on study design and animal behaviour was provided by RES. All authors have read and approved the final manuscript.

## References

[B1] Brown WV Fujioka K Wilson PW Woodworth KA Obesity: why be concerned? Am J Med 2009 122 Suppl 1 S4 11 19410676 10.1016/j.amjmed.2009.01.002

[B2] Kirk EP Klein S Pathogenesis and pathophysiology of the cardiometabolic syndrome J Clin Hypertens 2009 11 761 765 10.1111/j.1559-4572.2009.00054.x PMC2859214 20021538

[B3] Wannamethee SG Shaper AG Lennon L Morris RW Metabolic syndrome vs Framingham Risk Score for prediction of coronary heart disease, stroke, and type 2 diabetes mellitus Arch Intern Med 2005 165 2644 50 10.1001/archinte.165.22.2644 16344423

[B4] Bray GA Nielsen SJ Popkin BM Consumption of high-fructose corn syrup may play a role in the epidemic of obesity Am J Clin Nutr 2004 79 537 543 15051594 10.1093/ajcn/79.4.537

[B5] Vartanian LR Schwartz MB Brownell KD Effects of soft drink consumption on nutrition and health: a systematic review and meta-analysis Am J Pub Health 2007 97 667 675 10.2105/AJPH.2005.083782 PMC1829363 17329656

[B6] Deshmukh-Taskar PR O'Niel CE Nicklas TA Yang S-J Liu Y Gustat J Berenson GS Dietary patterns associated with metabolic syndrome, sociodemographic and lifestyle factors in young adults: the Bogalusa Heart Study Public Health Nutr 2009 12 2493 2503 10.1017/S1368980009991261 19744354 PMC2792890

[B7] Cohen DA Sturm R Scott M Farley TA Bluthenthal R Not enough fruit and vegetables or too many cookies, candies, salty snacks, and soft drinks? Public Health Rep 2010 125 88 95 20402200 10.1177/003335491012500112 PMC2789820

[B8] Kanarek RB Hirsch E Dietary-induced overeating in experimental animals Fed Proc 1977 36 154 158 838081

[B9] VandeBerg JL VandeBerg JL, Williams-Blangero S, Tardif SD Introduction The baboon in biomedical research 2009 New York, Springer xvii xxiii

[B10] Crossley JN MacDonald I The influence in male baboons, of a high sucrose diet on portal and arterial levels of glucose and fructose following a sucrose meal Nutr Metabol 1970 12 171 178 10.1159/000175290 4990715

[B11] Jolly CJ A proper study for mankind: anologies from the Papionin monkeys and their implications for human evolution Yrbk Phys Anthropol 2001 44 177 204 10.1002/ajpa.10021 11786995

[B12] Codron D Lee-Thorp JA Sponheimer M de Ruiter D Codron J Inter- and intra-habitat dietary variability of Chacma baboons (Papio ursinus) in South African savannas based on fecal δ^13^C, δ^15^N, and %N Am J Phys Anthropol 2006 129 204 214 10.1002/ajpa.20253 16247809

[B13] Kemnitz JW Sapolsky RM Altmann J Muruthi P Mott GE Stefanick ML Effects of food availability on serum insulin and lipid concentrations in free-ranging baboons Am J Primatol 2002 57 13 19 10.1002/ajp.1083 11977122

[B14] Banks WA Altmann J Sapolsky RM Phillips-Conroy JE Morley JE Serum leptin levels as a marker for syndrome X-like condition in wild baboons J Clin Endocrinol Metab 2003 88 1234 1240 10.1210/jc.2002-021695 12629112

[B15] Comuzzie AG Cole SA Martin L Carey KD Mahaney MC Blangero J VandeBerg JL The baboon as a nonhuman primate model for the study of the genetics of obesity Obesity 2003 11 75 80 10.1038/oby.2003.12 12529488

[B16] Chavez AO Lopez-Alvarenga JC Tejero ME Triplitt C Bastarrachea RA Sriwijitkamol A Tantiwong P Voruganti VS Musi N Comuzzie AG DeFronzo RA Folli F Physiological and molecular determinants of insulin action in the baboon Diabetes 2008 57 899 908 10.2337/db07-0790 18174524

[B17] Guardado-Mendoza R Davalli AM Chavez AO Hubbard GB Dick EJ Majluf-Cruz A Tene-Perez CE Goldschmidt L Hart J Perego C Comuzzie AG Tejero ME Finzi G Placidi C La Rosa S Capella C Halff G Gastaldelli A DeFronzo RA Folli F Pancreatic islet amyloidosis, beta-cell apoptosis, and alpha-cell proliferation are determinants of islet remodeling in type-2 diabetic baboons Proc Natl Acad Sci USA 2009 106 13992 13997 10.1073/pnas.0906471106 19666551 PMC2729008

[B18] Tejero ME Freeland-Graves JH Proffitt JM Peebles KW Cai G Cole SA Comuzzie AG Adiponectin but not resistin is associated with insulin resistance-related phenotypes in baboons Obes Res 2004 12 871 877 10.1038/oby.2004.105 15166309

[B19] Chavez AO Gastaldelli A Guardado-Mendoza R Lopez-Alvarenga JC Leland MM Tejero ME Sorice G Casiraghi F Davalli A Bastarrachea RA Comuzzie AG DeFronzo RA Folli F Predictive models of insulin resistance derived from simple morphometric and biochemical indices related to obesity and the metabolic syndrome in baboons Cardiovasc Diabetol 2009 8 22 10.1186/1475-2840-8-22 19389241 PMC2674590

[B20] Rainwater DL Cox LA Rogers J VandeBerg JL Mahaney MC Localization of multiple pleiotropic genes for lipoprotein metabolism in baboons J Lipid Res 2009 50 1420 8 10.1194/jlr.M800583-JLR200 19270339 PMC2694340

[B21] Strong JP McGill HC Diet and experimental atherosclerosis in baboons Experimental Atherosclerosis 1967 50 669 690 PMC1965309 4960563

[B22] Kushwaha R McGill HC Diet, plasma lipoproteins, and experimental atherosclerosis in baboons (*Papio Sp*) Human Reproduction Update 1998 4 420 429 10.1093/humupd/4.4.420 9825856

[B23] Kritchevsky D Davidson LM Shapiro IL Kim HK Kitagawa M Malhotra S Nair PP Clarkson TB Bersohn I Winter PAD Lipid metabolism and experimental atherosclerosis in baboons: influence of cholesterol-free, semi-synthetic diets Am J Clin Nutr 1974 27 29 50 4358391 10.1093/ajcn/27.1.29

[B24] Miyazaki Y DeFronzo RA Visceral fat dominant distribution in male type 2 diabeteic patients is closely related to hepatic insulin resistance, irrespective of body type Cardiovasc Diabetol 2009 8 44 10.1186/1475-2840-8-44 19656356 PMC2729732

[B25] Kim SP Ellmerer M Van Citters GW Bergman RN Primacy of hepatic insulin resistance in the development of the metabolic syndrome induced by an isocaloric moderate-fat diet in the dog Diabetes 2003 52 2453 2460 10.2337/diabetes.52.10.2453 14514627

[B26] Forbes GB Brown MR Welle SL Lipinski BA Deliberate overfeeding in women and men: energy cost and composition of weight gain Br J Nutr 1986 56 1 9 10.1079/BJN19860080 3479191

[B27] Forbes GB Lean body mass-body fat interrelationships in humans Nutr Rev 1987 45 225 231 10.1111/j.1753-4887.1987.tb02684.x 3306482

[B28] Norgan NG Durnin JV The effect of 6 weeks of overfeeding on the body weight, body composition, and energy metabolism of young men Am J Clin Nutr 1980 33 978 988 7369169 10.1093/ajcn/33.5.978

[B29] Lehmann R Wagner JL Fernandez LA Bourgoignie JJ Ricordi C Alejandro R Kenyon NS Effects of ketamine sedation on glucose clearance, insulin secretion, and counterregulatory hormone production in baboons (Papio hamadryas) J Med Primatol 1997 26 312 321 9438225 10.1111/j.1600-0684.1997.tb00060.x

[B30] Hotta K Funahashi T Bodkin NL Ortmeyer HK Arita Y Hansen BC Matsuzawa Y Circulating concentrations of the adipocyte protein adiponectin are decreased in parallel with reduced insulin sensitivity during progression to type 2 diabetes in rhesus monkeys Diabetes 2001 50 1126 1133 10.2337/diabetes.50.5.1126 11334417

[B31] McGill HC Mcmahan A Kruski AW Kelley JL Mott GE Responses of serum lipoproteins to dietary cholesterol and type of fat in the baboon Arteriosclerosis 1981 5 337 344 10.1161/01.atv.1.5.337 6956267

[B32] Bantle JP Raatz SK Thomas W Georgopoulos A Effects of dietary fructose on plasma lipids in healthy subjects Am J Clin Nutr 2000 72 1128 1134 11063439 10.1093/ajcn/72.5.1128

[B33] Adochino RL Leitner JW Gray K Draznin B Cornier MA Early responses of insulin signaling to high-carbohydrate and high-fat overfeeding Nutrition & Metabolism 2009 6 37 10.1186/1743-7075-6-37 PMC2761378 19781106

[B34] Minehira K Bettschart V Vidal H Vega N Di Vetta V Rey V Schneiter P Tappy L Effect of carbohydrate overfeeding on whole body and adipose tissue metabolism in humans Obes Res 2003 11 1096 1103 10.1038/oby.2003.150 12972680

[B35] McDevitt RM bott SJ Harding M Coward WA Bluck LJ Prentice AM De novo lipogenesis during controlled overfeeding with sucrose or glucose in lean and obese women Am J Clin Nutr 2001 74 737 746 11722954 10.1093/ajcn/74.6.737

[B36] Brons C Jensen CB Storgaard H Hiscock NJ White A Appel JS Jacobsen S Nilsson E Larsen CM Astrup A Quistorff B Vaag A Impact of short-term high-fat feeding on glucose and insulin metabolism in young healthy men J Physiol 2009 587 2387 2397 10.1113/jphysiol.2009.169078 19332493 PMC2697306

[B37] Del Mar Romero M Fernandez-Lopez JA Esteve M Alemany M Different modulation by dietary restriction of adipokine expression in white adipose tissue sites in the rat Cardiovasc Diabetol 2009 8 42 10.1186/1475-2840-8-42 19642981 PMC3224727

[B38] Wang J Obici S Morgan K Barzilai N Feng Z Rossetti L Overfeeding rapidly induces leptin and insulin resistance Diabetes 2001 50 2786 2791 10.2337/diabetes.50.12.2786 11723062

[B39] Mooradian AD Chehade J Hurd R Haas MJ Monosaccharide-enriched diets cause hyperleptinemia without hypophagia Nutrition 2000 16 439 441 10.1016/S0899-9007(00)00229-X 10869900

[B40] Ukkola O Teran-Garcia M Tremblay A Despres JP Bouchard C Adiponectin concentration and insulin indicators following overfeeding in identical twins J Endocrinol Invest 2008 31 132 137 18362504 10.1007/BF03345579

[B41] Le K-A Faeh D Stettler R Ith M Kreis R Vermathen P Boesch C Ravussin E Tappy L A 4-wk high fructose diet alters lipid metabolism without affecting insulin sensitivity or ectopic lipids in health humans Am J Clin Nutr 2006 84 1374 1379 17158419 10.1093/ajcn/84.6.1374

[B42] Hansen BC Pathophysiology of obesity-associated type II diabetes (NIDDM): Implications from longitudinal studies of non-human primates Nutrition 1989 5 48 50 2520257

